# Effects of oral treatment with chondroitin sulfate and glucosamine in an experimental model of metacarpophalangeal osteoarthritis in horses

**DOI:** 10.1186/s12917-022-03323-3

**Published:** 2022-06-09

**Authors:** Ana Lucia Miluzzi Yamada, Cynthia do Prado Vendruscolo, Marília Ferrari Marsiglia, Eric Danilo Pauls Sotelo, Fernanda Rodrigues Agreste, Sarah Raphaela Torquato Seidel, Joice Fülber, Raquel Yvonne Arantes Baccarin, Luis Claudio Lopes Correia da Silva

**Affiliations:** 1 Department of Surgery, School of Veterinary Medicine and Animal Science, University of São Paulo, Av. Prof. Dr. Orlando Marques de Paiva, 87. Cidade Universitária, São Paulo, SP CEP: 05508-270 Brazil; 2 Department of Internal Medicine, School of Veterinary Medicine and Animal Science, University of São Paulo, Av. Prof. Dr. Orlando Marques de Paiva, 87. Cidade Universitária, São Paulo, SP CEP: 05508-270 Brazil

**Keywords:** Equine, Glycosaminoglycan, Joint, Lameness, Osteoarthritis

## Abstract

**Background:**

Combined chondroitin sulfate (CS) and glucosamine (GlcN) has been widely used in oral formulations to prevent and treat osteoarthritis. CS is effective for controlling pain in osteoarthritic patients, whereas GlcN can stimulate glycosaminoglycan synthesis, thus reducing extracellular matrix degradation. Although several studies have been published on this topic, the effectiveness of treatment with oral CS and GlcN remains uncertain. The objective of this study was to analyze the progression of experimentally induced osteoarthritis in horses and verify the effectiveness of an oral compound based on CS and GlcN to treat and/or modulate this disease. The study analyzed the metacarpophalangeal joint of the left thoracic limb of 16 horses divided into two groups, with eight horses treated with CS and GlcN in the treated group (GT) and eight untreated horses in the control group (GC). Chondral lesions were induced through arthroscopy, which was defined as time-point zero (T0). Physical, ultrasonographic, and radiographic examinations and synovial fluid biomarkers measurements were performed on days 0, 30, 60, 90, and 120. At the end of the experiment (T4), arthroscopy was performed again to macroscopically evaluate the joints and collect material for microscopic analysis.

**Results:**

Significant differences were observed between groups in some evaluated parameters, such as visual lameness assessment, synovial concentrations of prostaglandin E2, and ultrasound examination. However, the GT still presented slightly improved results for joint flexion angle, analysis of lameness using sensors, and histopathological analysis of chondral repair tissue, however, without the statistical significance (*p*>0.05).

**Conclusions:**

The treatment was considered effective in the clinical modulation of experimental osteoarthritis, with improvement of some parameters in the GT. However, this type of treatment may not be entirely effective to change the catabolic process in articular cartilage and the progressive induced chondral damage.

## Background

Osteoarthritis is a major cause of lameness in horses. It is a degenerative disease that leads to severe morpho-functional disability and consequent sports retirement. Currently, osteoarthritis is characterized by a conglomerate of overlapping disorders of different etiologies, but with a common outcome: the progressive deterioration of osteochondral tissue [1, 2]. This disease also involves all joint structures, including the capsule, synovial membrane, ligaments, and periarticular tissues; however, progressive deterioration of the subchondral bone and cartilage is the main consequence of osteoarthritis [3]. In most cases, the horse presents with joint pain, lameness, and physical disability. This group of disorders resulting from osteoarthritis makes the treatment challenging, especially in athletes. Therefore, prevention and monitoring measures are essential to maintain joint integrity in these horses [3–6].

Chondroitin sulfate (CS) is one of the main components of the extracellular matrix of articular cartilage and plays an important role in skeletal formation, being essential for proper functioning of the joints [7]. Some studies have reported an advantageous association of CS with glucosamine (GlcN) to treat and modulate osteoarthritis owing to an important chondroprotective effect [8–10].

The oral administration of CS is well tolerated and effective in controlling pain in osteoarthritic patients, especially at higher dosages [11]. It can reduce the concentration of catabolic mediators and pro-inflammatory cytokines, it also suppresses others inflammatory mediators and the tissue degradation [8, 12, 13]. The chondroitin sulfate prevents chondral degeneration by inhibiting hydrolytic and proteolytic enzymes, reducing the action of collagenase, attenuating oxidative events, and consequently reducing the progression of osteoarthritis. CS is characterized by its low cost and excellent safety and can be used in both the treatment and prevention of osteoarthritis [11, 12].

The combination of CS and GlcN has shown positive results in several studies [8–10, 14]. Glucosamine is an aminosugar that is present in the glycosaminoglycans structure. The GlcN also contributes to maintaining flexibility, shock absorption, and tissue resistance [8]. Recent studies have shown that GlcN can stimulate the synthesis of glycosaminoglycans, decreasing extracellular matrix degradation and reducing the progression of osteoarthritis [15]. GlcN also has significant therapeutic potential in the treatment of osteoarthritis, being equally capable of reducing intra-articular inflammation and stimulating the synthesis of type II collagen [16].

Although several studies have been published on this topic, the effectiveness of treatment with oral CS and GlcN remains uncertain. Some authors suggest that there is no adequate systemic distribution or significant improvement to support the use of these products [17]. However, there is also report that this combination can be especially useful in preventing and treating osteoarthritis, even working as a disease-modifying osteoarthritis drug (DMOAD) [8]. Thus, randomized and controlled experimental studies are essential to prove the possible chondroprotective action and effectiveness of these oral therapeutic compounds by following up the progress of osteoarthritis and the treatment effect. Therefore, the objective of this study was to analyze the progression of osteoarthritis experimentally induced in horses and verify the effectiveness of an oral compound based on CS and GlcN to treat and/or modulate the disease.

## Results

No preexisting lesions were identified in any of the metacarpophalangeal joints on physical, ultrasonographic, and radiographic examinations at T0 or during the experimental induction of osteoarthritis, allowing the immediate inclusion of all mares in the experiment. No horses were excluded from study.

**Fig. 1:**
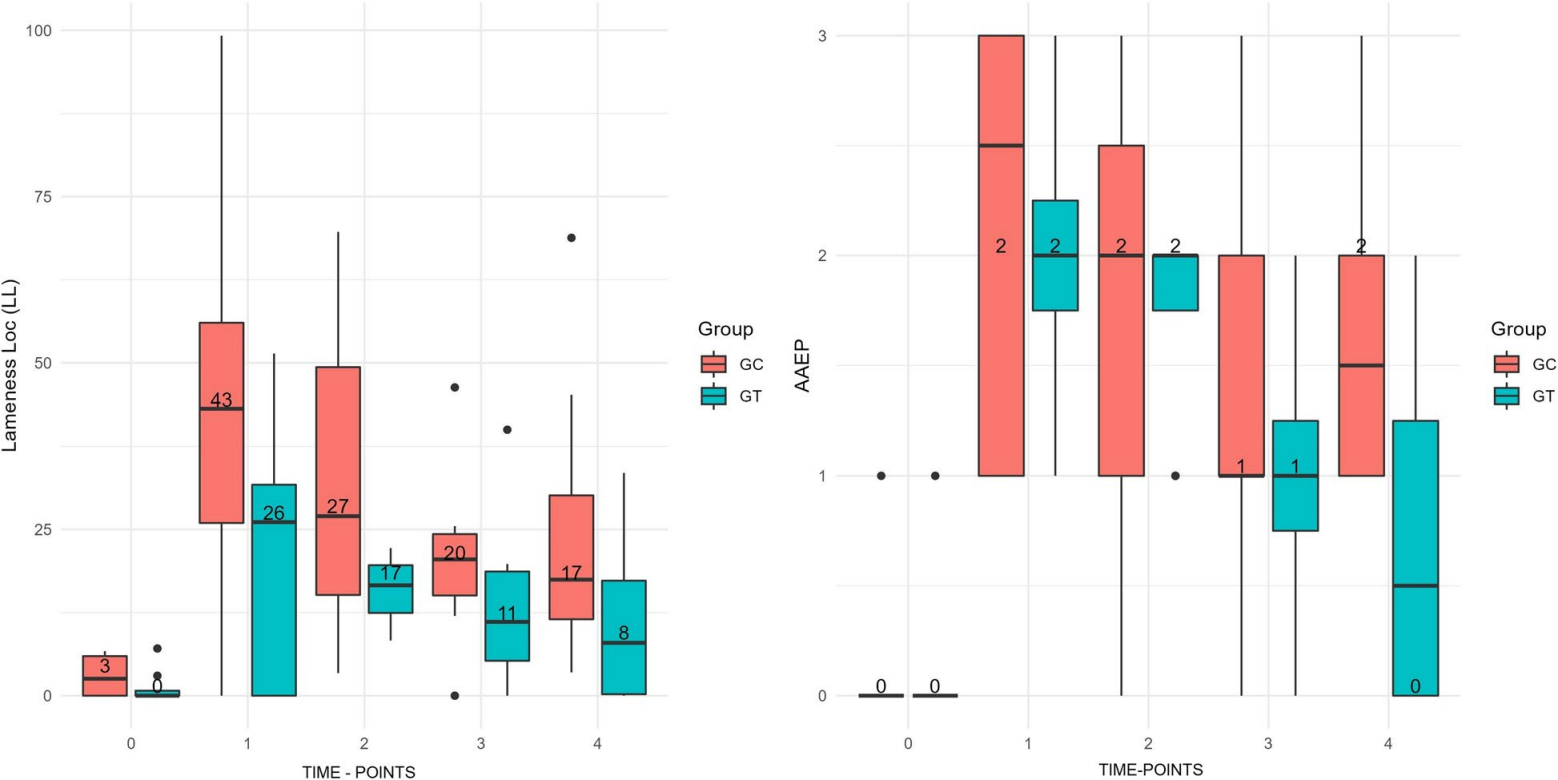
Lameness assessment: Boxplot graphs were applied when data were not normally distributed, and the best measure of central tendency was the median. The scores were obtained after the lameness evaluation using the American Association of Equine Practitioners lameness scale (AAEP) and analysis using the motion sensor (Lameness Locator), at times T0 to T4, for the control group (GC) and treated group (GT). The boxes represent data variability. The bottom of the box corresponds to the point of 25% of the sample, the top of the box corresponds to the point of 75% of the sample, and the line inside corresponds to the median (point that divides the sample by 50%, in the center of distribution). *Presence of outliers, there was statistical difference between groups at T4 (*p* < 0.05)

The results of visual lameness assessment (AAEP Lameness Scale) and the evaluation by motion sensors (Lameness Locator) are shown in Table 1 and Fig. 1. There was a difference between groups in the visual lameness test using the AAEP scores, with statistical significance at T4 (p < 0.05). The examination using movement sensors showed an interesting difference between groups. The GT increased the Q-score from 1 at T0 to 22 at T1 while the GC increased the Q-score from 3 at T0 to 44 at T1, however, this difference between groups was not statistically significant.

**Table 1:** Joint angles (in degrees), visual lameness (AAEP scale) and evaluation by motion sensors (Lameness Locator) measured from T0 to T4 in the GC and GT

		T0	T1	T2	T3	T4	***p***-value, Friedman test between time-points	***p***-value, ANOVA^**a**^
	**Joint angles**							
**GC**	Mean ± SD	138.8 ± 5.9	149 ± 3.9	150.6 ± 5.3	148.3 ± 6.5	149.5 ± 5.3	**0.004**	**0.158**
	Median (IQR)	140.5 (133.5–143)	148.5 (146.5–149)	151.5 (146–154.5)	150.5 (142.5–153)	150 (146.5–153)		
**GT**	Mean ± SD	141.8 ± 6.2	148.8 ± 5.3	147 ± 5.1	145.4 ± 2.1	148.1 ± 2.1	**0.005**	
	Median (IQR)	143.5 (140–146)	149.5 (147–151.5)	148 (143.5–150)	146 (144.5–146.5)	147.5 (146.5–149.5)		
	**Lameness Locator**							
**GC**	Mean ± SD	3 ± 3	44 ± 32	33 ± 25	21 ± 13	25 ± 22	**0,015**	**0,793**
	Median (IQR)	3 (0–6)	44 (22–62)	27 (14–59)	21 (14–25)	18 (11–35)		
**GT**	Mean ± SD	1 ± 3	22 ± 20	16 ± 5	13 ± 13	11 ± 12	**0,001**	
	Median (IQR)	0 (0–2)	26 (0–35)	17 (12–21)	11 (4–19)	8 (0–19)		
	**AAEP score**							
**GC**	Mean ± SD	0 ± 0	2 ± 1	2 ± 1	1 ± 1	2 ± 1	**0,011**	
	Median (IQR)	0 (0–0)	3 (1–3)	2 (0–3)	1 (1–2)	2 (1–2)		**0,169**
**GT**	Mean ± SD	0 ± 0	2 ± 1	2 ± 0	1 ± 1	1 ± 1	**< 0,001**	
	Median (IQR)	0 (0–0)	3 (1–3)	2 (0–3)	1 (1–2)	2 (1–2)		
***p*-value Mann-Whitney U test between groups at each time-point**								
	Lameness Locator	0,382	0,13	0,232	0,195	0,13		
	AAEP score	1	0,798	0,779	0,505	0,038		

*ANOVA* analysis of variance, *GC* control group, *SD* standard deviation, *GT* treated group

^a^*P*-value of time-point/group interaction by nonparametric two-way ANOVA

The results of the comparison between groups regarding the joint angle are shown in Table 1. The GT presented a mean measurement of the maximum joint flexion angle lower than the GC, reaching a difference of more than 3° at T2. These data show that the ability to flex the joint in the GT was better preserved, with a difference between T0 and T4 of 6.3° in the GT and 10.7° in the GC. However, this difference between groups was not statistically significant (*p = 0.158*).

The mean results of the comparison between groups for the biomarkers CTX II and PGE2 and for the quantification of glycosaminoglycans are shown in Table 2. The biomarker CTX II showed a significant difference among evaluation time-points, but not between groups. There were no statistically significant differences in CS and hyaluronic acid biomarkers (glycosaminoglycans) between time-points or groups. The joints responded to the experimental chondral damage between time-points, especially after T1. CTX II values significantly increased until T4, with a statistically significant difference between T0 (mean of 261.75 pg/mL) and T4 (mean of 605.65 pg/mL) in both groups (*p* < 0.001). As expected, osteoarthritis induction was confirmed by the increasing levels of this biomarker in the synovial fluid. PGE2 showed a statistically significant difference between time-points only in the GC (*p* < 0.05).

**Table 2:** Mean concentrations of biomarkers in synovial fluid, measured from T0 to T4 in the GC and GT

Biomarker		T0	T1	T2	T3	T4	***P***-value, Friedman test between time-points	***P***-value ANOVA^a^
**CTXII (pg/mL)**								**0.568**
GC	Mean ± SD	255.2 ± 38.9	210.6 ± 95.4	386,5 ± 188	593.8 ± 51.5	593.2 ± 66.1	**0.003**	
	Median (IQR)	260.5 (223.7–285.5)	172.3 (154.4–231.8)	370.3 (226.1–538.6)	619.3 (547.9–629.3)	586.1 (541.9–649.9)		
GT	Mean ± SD	268.3 ± 42	238 ± 61.1	429.2 ± 201.9	566.3 ± 104.6	618.1 ± 110.1	**< 0.001**	
	Median (IQR)	268.1 (231.3–297.3)	231.3 (211–264.8)	433 (236.5–622.2)	547.9 (489.3–607.2)	617.9 (543.6–640.7)		
**PGE2 (pg/mL)**								**0.078**
GC	Mean ± SD	25.5 ± 10.7	82.6 ± 40.4	75 ± 38.4	47.4 ± 27.5	50.9 ± 29.4	**0.004**	
	Median (IQR)	21.3 (17.2–33.3)	81.8 (48.6–122.4)	65.4 (42–116.5)	34.1 (31.4–59.8)	46 (26–81.3)		
GT	Mean ± SD	47.2 ± 40.7	60.7 ± 32.2	55.8 ± 47.4	45.9 ± 46.2	60.1 ± 38	**0.171**	
	Median (IQR)	26.8 (19–77.5)	53.7 (38.1–73.7)	41.9 (31.9–57.3)	24.4 (17.9–67.7)	51.2 (34.6–81.4)		
**Chondroitin sulfate (μg/mL)**								**0.318**
GC	Mean ± SD	23.5 ± 9.5	26.4 ± 10.6	25.4 ± 16	31.6 ± 12.7	28.8 ± 8.4	**0.711**	
	Median (IQR)	21.2 (15.7–29.8)	25.5 (18.6–29.3)	18 (13.6–35.4)	28.4 (25.7–31.7)	29 (22.2–36.6)		
GT	Mean ± SD	29.4 ± 8.1	20.7 ± 5	24.2 ± 10.7	23.9 ± 18.4	34.7 ± 15.7	**0.103**	
	Median (IQR)	27.9 (24.9–33.3)	19.5 (16.9–24.3)	20.9 (17–30.7)	15.1 (13.3–29.8)	33 (22.2–44.2)		
**Hyaluronic acid (μg/mL)**								**0.517**
GC	Mean ± SD	556.5 ± 110.6	301.6 ± 113.6	365.2 ± 122.8	310 ± 120.6	381.5 ± 127.7	**0.034**	
	Median (IQR)	574.9 (473.5–600.6)	297.7 (216–383.7)	372.5 (243.5–484.1)	328.5 (207–419.9)	409.4 (297.5–466.4)		
GT	Mean ± SD	579.9 ± 75.3	304.8 ± 91.6	388.7 ± 168.2	392.1 ± 146.6	471.5 ± 107	**0.001**	
	Median (IQR)	591.2 (504–641.8)	321.4 (222–370.5)	337.3 (248.6–534.8)	405.1 (319.6–508.2)	518 (392.9–550.6)		
**p-value Mann-Whitney U test between groups at each time-point**								
**CTXII (pg/mL)**		0,798	0,234	0,505	0,414	0,779		
**PGE2 (pg/mL)**		0,279	0,336	0,281	0,181	0,731		
**Chondroitin sulfate (μg/mL)**		0,161	0,234	0,613	0,121	0,645		
**Hyaluronic acid (μg/mL)**		0,574	0,878	0,867	0,281	0,130		

*ANOVA* analysis of variance, *GC* control group, *CTX II* C-telopeptide of type II Collagen, *PGE2* prostaglandin E2, *SD* standard deviation, *GT* treated group

^a^*P*-value of time-point/group interaction by nonparametric two-way ANOVA

**Fig. 2:**
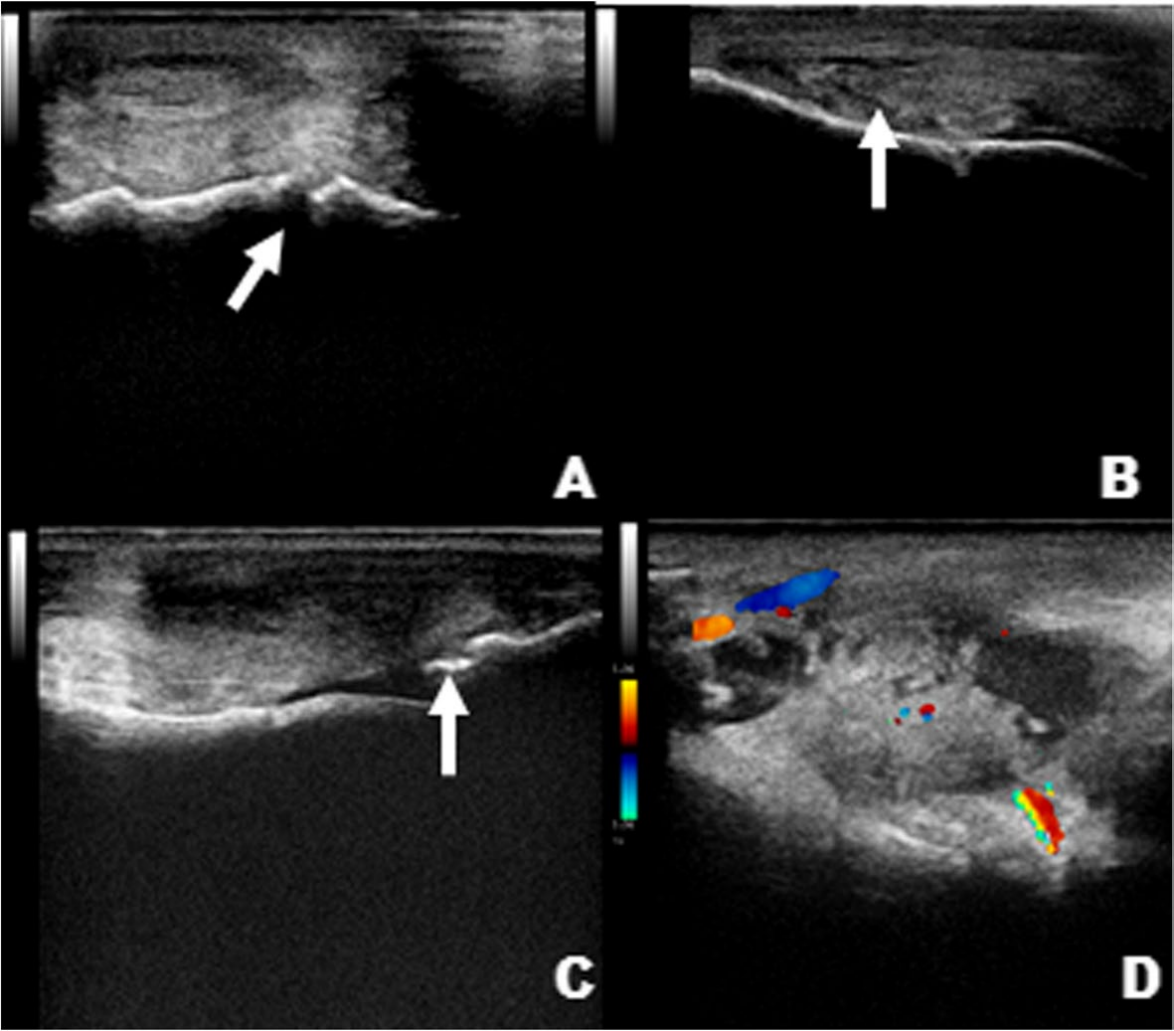
Ultrasonographic images of GC (**C** and **D**) and GT (**A** and **B**): the images show osteochondral irregularities on the condyles (arrow in **A**), enlarged and heterogeneous plica (arrow in **B**), osteophytes and fragmentation in the first phalanx (arrow in **C**), and synovitis with increased synovial vascularization (color Doppler in **D**)

The results of the ultrasonographic evaluation (mean sum of the scores – Table 3) are shown in Table 4. The most frequently observed types of injuries were the same for both groups, including mainly synovitis, increased synovial vascularization, osteochondral irregularities in the condyles and first phalanx, increased and heterogeneous synovial plica, and irregularities in periarticular ligaments and osteophytes (Fig. 2). However, the GC received higher scores. Joint deterioration became evident soon after the arthroscopy at T0, with evident changes at T1, and progressively increasing until T4. The GT had a lower score on ultrasonographic examination, especially at T4, than did the GC, which was statistically different at this time (11 points in the GC and 7 in the GT) (*p* < 0.001).

**Table 3:** Predefined scores [18] for the ultrasonographic evaluation of images obtained at time-points T0 to T4

Ultrasound characteristics	Score
**Synovial fluid appearance**	
Normal (anechoic)	0
Slightly changed (predominantly anechoic/rare hyperechogenic spots)	1
Changed (heterogeneous liquid with anechoic points)	2
Presence of fibrin/evident hyperechogenic spots, predominantly heterogeneous	3
**Synovial fluid quantity**	
Normal	0
Slightly increased	1
Increased	2
Severe discharge	3
**Appearance of the joint capsule (thickness and insertions)**	
Normal	0
Slightly changed (noticeable thickening and heterogeneity)	1
Changed (clearly thickened and heterogeneous/few proliferations at insertion)	2
Severely changed (thickened and heterogeneous, with calcification points and/or intense proliferation at insertion)	3
**Appearance of periarticular ligaments (origin and insertions)**	
Normal	0
Slightly changed (noticeable heterogeneity)	1
Changed (clearly heterogeneous and with proliferations at origin/insertion)	2
Severely changed	3
**Vascularization and synovitis**	
None	0
Mild (few vessels visible, synovium slightly hypertrophied)	1
Moderate (evident vascularity, congestion and hypertrophied synovium)	2
Severe	3
**Appearance of articular cartilage**	
Normal	0
Slightly irregular	1
Discontinuous and rough	2
Difficult to visualize, erosions, fragments	3
**Subchondral surface**	
Normal	0
Irregular with focal lesions	1
Clearly irregular	2
Depressions, erosions, fragments	3
**Appearance of the plica**	
Normal	0
Mildly changed	1
Changed (heterogeneous and hypertrophied)	2
Severely changed	3
**Presence of osteophytes**	
None	0
Mild projection	1
Evident projection	2
Severe projection	3
**Final sum (total)**	27

**Table 4:** Scores of ultrasonographic and radiographic evaluations (mean of the sum of the scores as shown in Tables 3 and 5) from T0 to T4 in the GC and GT

		T0	T1	T2	T3	T4	***P***-value, Friedman test between time-points	***P***-value ANOVA^a^
**Ultrasonographic**								
GC	Mean ± SD	2 ± 2	6 ± 3	6 ± 3	8 ± 3	11 ± 3	0,001	
	Median (IQR)	2 (2–4)	5 (4–8)	6 (5–7)	7 (6–10)	10 (9–14)		**0,564**
GT	Mean ± SD	2 ± 2	5 ± 3	6 ± 2	6 ± 1	7 ± 1	0,003	
	Median (IQR)	2 (0–3)	5 (3–6)	6 (4–7)	6 (6–7)	7 (6–8)		
**Radiographic**								
GC	Mean ± SD	1 ± 1	3 ± 1	3 ± 1	2 ± 1	3 ± 2	0,005	**0,361**
	Median (IQR)	0 (0–1)	3 (2–4)	3 (2–3)	2 (2–3)	3 (2–4)		
GT	Mean ± SD	2 ± 1	3 ± 1	2 ± 1	2 ± 1	2 ± 1	0,038	
	Median (IQR)	1 (1–2)	3 (2–4)	2 (1–2)	2 (1–2)	2 (2–3)		
***p*-value Mann-Whitney U test between groups at each time-point**								
	Ultrasonographic	0,645	0,645	0,798	0,336	< 0,001		
	Radiographic	0,051	0,79	0,123	0,872	0,597		

*ANOVA* analysis of variance, *GC* control group, *SD* standard deviation, *GT* treated group

^a^*P*-value of time-points/group interaction by nonparametric two-way ANOVA

Minor radiographic changes were observed especially after T3 (mean sum of the scores – Table 5). Both the GT and the GC presented mainly increased soft tissue volume especially at T1, osteophyte and enthesophyte formation, bone proliferation, areas of osteolysis, decreased radiographic interline, and rare fragmentations. The radiographic examinations showed no significant differences between groups (*p* > 0.05) (Table 4).

**Table 5:** Predefined scores [6] for the radiographic evaluation of images obtained at time-points T0 to T4

Radiograph characteristics	Score
**Increased volume of soft tissues**	
None	0
Mild	1
Moderate	2
Severe	3
**Presence of soft tissue mineralization**	
None	0
Suspected	1
Evident	2
Severe	3
**Increased radiographic interline**	
None	0
Suspected	1
Evident	2
Severe	3
**Decreased radiographic interline**	
None	0
Suspected	1
Evident	2
Severe	3
**Presence of osteophytes and bone proliferations**	
None	0
Mild	1
Moderate	2
Severe	3
**Presence of enthesophytes**	
None	0
Mild	1
Moderate	2
Severe	3
**Presence of subchondral sclerosis**	
None	0
Suspected	1
Evident	2
Severe	3
**Presence of subchondral osteolysis**	
None	0
Suspected	1
Evident	2
Severe	3
**Osteochondral fragments**	
None	0
One	1
Two	2
Multiple, on-site or displaced	3
**Final sum (total)**	27

**Fig. 3:**
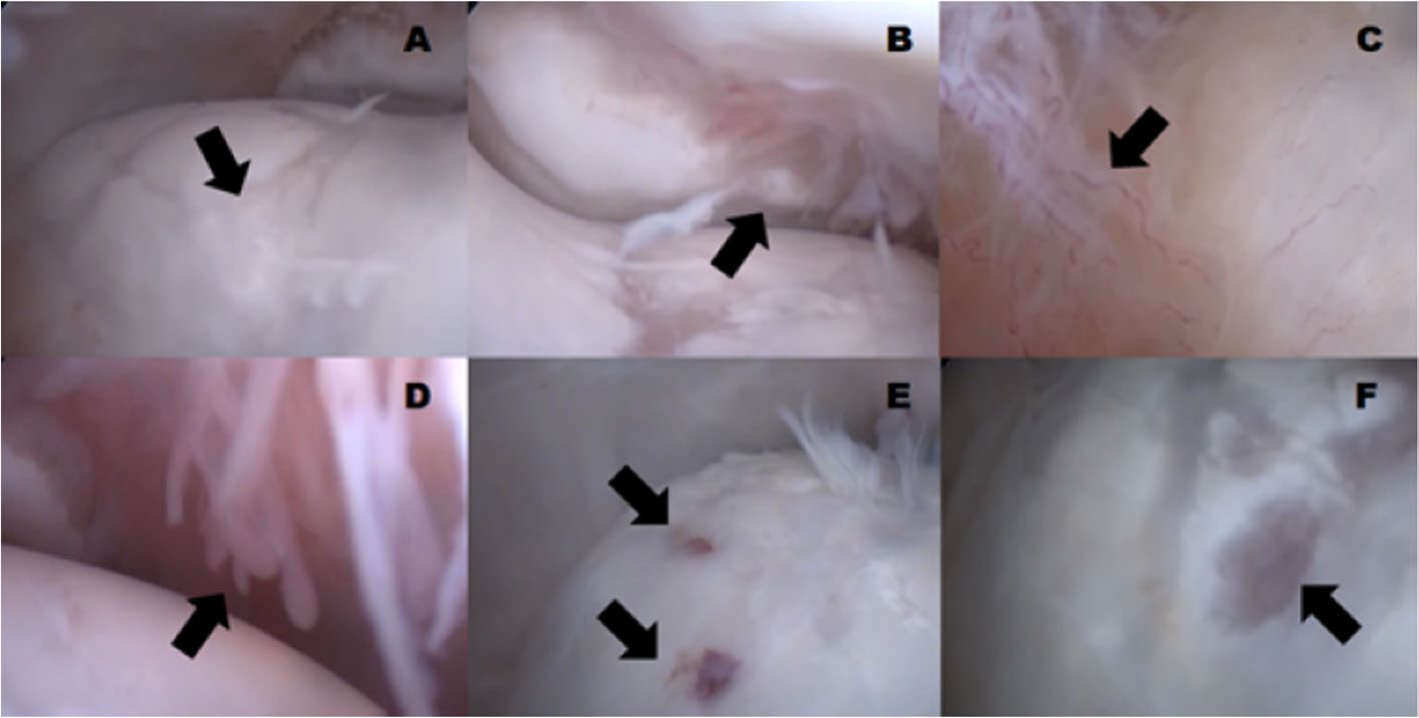
Arthroscopic images: the images at T4 show fibrocartilage formation at the lesion site (arrow in **A**), osteophytes and fragmentations in the first phalanx (arrow in **B**), increased vascularization and villous hypertrophy in synovitis (arrows in **C** and **D**), hemorrhage points and necrosis (arrows in **E**), and erosions (arrow in **F**)

Both groups showed fibrocartilage formation at the site of the chondral lesions, fibrillations, wear lines, erosions, necrosis points, synovitis, osteophytes, and fragmentations during the arthroscopic intervention and macroscopic assessment at T4 (Fig. 3). There was no significant difference between the GT and GC regarding the macroscopic arthroscopic scores of the joint at T4.

**Fig. 4:**
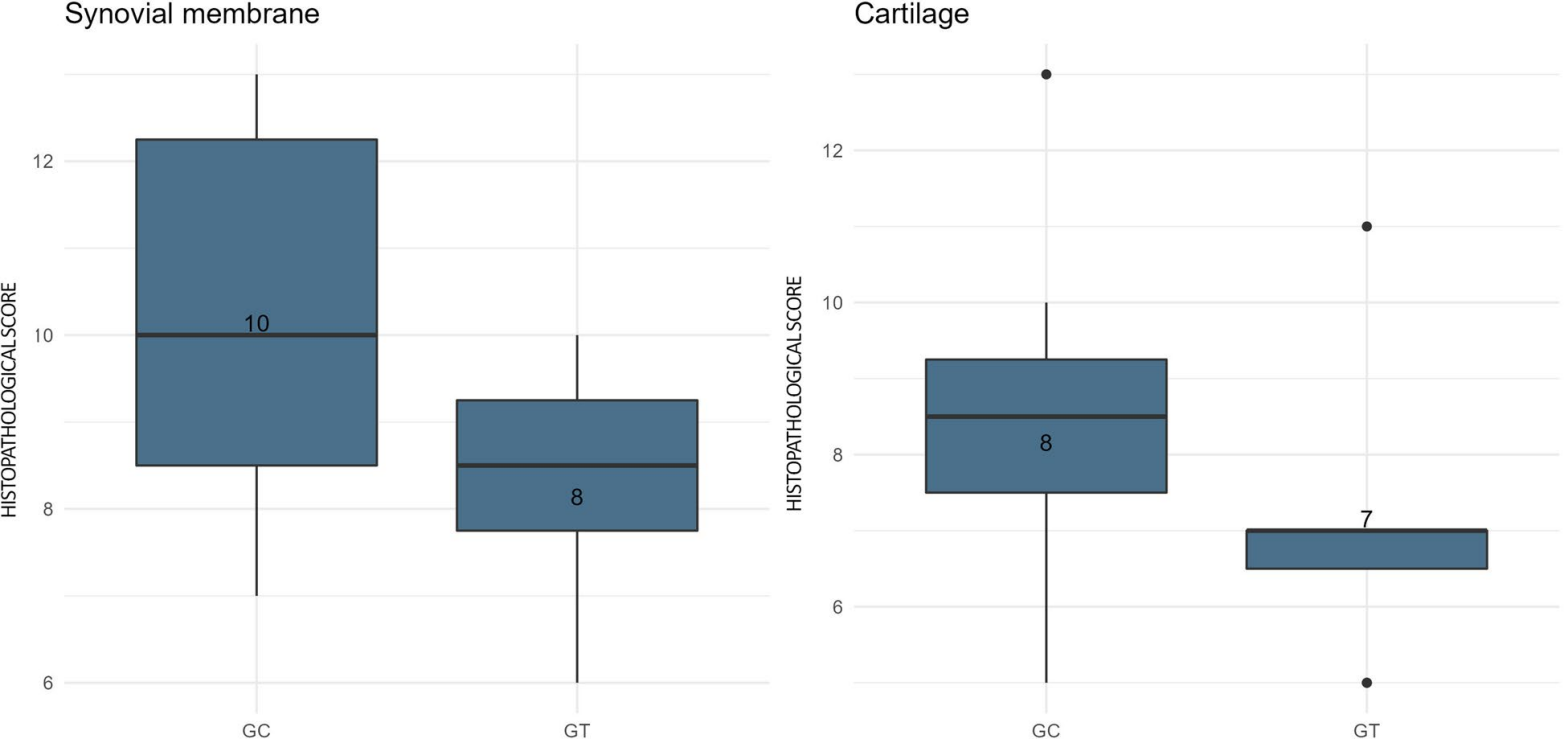
Microscopic analysis of the tissues at T4: Boxplot graphs were applied when data were not normally distributed, and the best measure of central tendency was the median. The graph shows the scores of the control group (GC) and treated group (GT) at T4 for synovial membrane and cartilage. The higher the score, the worse was the tissue condition. The boxes represent data variability. The bottom of the box corresponds to the point of 25% of the sample, the top of the box corresponds to the point of 75% of the sample, and the line inside corresponds to the median (point that divides the sample by 50%, in the center of distribution). *Presence of outliers. There was no statistically significant difference between the groups

The microscopic analysis of the tissues at T4 was limited to a small sample of cartilage and synovial membrane collected by arthroscopy at the site of T0 induction. Consequently, they may not have reflected the overall condition of tissues. The results at T4 are shown in Fig. 4 (mean sum of the scores – Table 6). The microscopic analyses of cartilage resulted in a lower score in the GT, but the difference was not statistically significant.

**Table 6:** Scores used in the histopathological analysis of synovial membrane and cartilage according to the osteoarthritis research society international [19]

Histopathological characteristics	Score	Description
**Synovial membrane**		
Cell infiltration (lymphocytes and polymorphonuclear cells)	0	None
	1	Occasional/in small areas
	2	Diffuse and mild presence
	3	Moderate presence
	4	Severe presence
Vascularization	0	Normal
	1	Mild focal increase
	2	Mild diffuse increase
	3	Moderate, focal or diffuse vessel enlargement or dilated vessels
	4	Severe increase in number or dilated vessels
Intimal hyperplasia	0	None
	1	Villi with 2–4 rows of intima cells within the section
	2	Villi with 4–5 rows of intima cells in 25–50% of the section
	3	Villi with 4–5 rows of intima cells in more than 25–50% of the section
	4	Villi with more than 5 rows of intima cells in more than 50% of the section
Subintimal edema	0	Without edema
	1	Mild edema
	2	Moderate edema
	3	Moderate edema in more than 50% of the section
	4	Severe diffuse edema
Degree of fibrosis	0	Normal
	1	Mild fibrosis
	2	Moderate and focal fibrosis
	3	Moderate and diffuse fibrosis in more than 50% of the section
	4	Severe and diffuse fibrosis
**Cartilage**		
Chondrocyte predominance and necrosis	0	Normal
	1	No more than one superficial necrotic cell per field at 20× magnification
	2	1–2 superficial necrotic cells per field at 20× magnification
	3	2–3 superficial necrotic cells per field at 20× magnification
	4	3–4 superficial necrotic cells per field at 20× magnification
Cluster formation	0	Normal
	1	Formation of superficial chondrocyte pairs
	2	Cluster formation of 2–3 chondrocytes
	3	Cluster formation of 3–4 chondrocytes
	4	Cluster formation of more than 4 chondrocytes in the section.
Cellularity loss	0	None
	1	10–20% of acellular area at 20× magnification
	2	20–30% of acellular area at 20× magnification
	3	40–50% acellular area at 20× magnification
	4	More than 50% of acellular area at 20× magnification
Fibrillation and surface fissures	0	None
	1	Restricted to the superficial area
	2	Extending to the middle zone
	3	Presence of fissures and fibrillations in the deep zone
	4	Severely changed deep zone
Fibrosis	0	Normal
	1	Mild
	2	Moderate and focal
	3	Moderate and diffuse in more than 50% of the section
	4	Severe and diffuse

## Discussion

Horses have been used and referred to as a valuable experimental model for the study of osteoarthritis with several therapeutic modalities and detailed monitoring of joint behavior [3, 20]. Equine medicine highlights the high incidence of metacarpophalangeal joint osteoarthritis, frequently showing signs of osteochondral impairment and degeneration, especially in athlete horses [3]. Controlled studies have reported the use of surgical induction of osteoarthritis as an experimental model frequently used to evaluate the effectiveness of different treatments [21–24]. Therefore, regarding the first objective of this study to analyze the progression of experimentally induced metacarpophalangeal joint osteoarthritis, the results were unambiguously favorable. Both groups showed a clear deterioration of the articular condition with evident lameness on visual and sensor examination, increased CTX II concentrations, progressively increased scores on ultrasonographic and radiographic examination, and macroscopic findings at T4 consistent with osteoarthritis and osteochondral degradation, which allowed the analysis of the joint response to treatment with a commercial compound.

As proposed in this study, it was decided just to evaluate the effectiveness of the combined CS and GlcN, the assessment of the separate CS and GlcN was not carried out. However, for the most part of studies in CS and GlcN effectiveness, the treatment uses the combination of these molecules and corroborating previously published reports [8, 9, 11, 12] positive results have also been demonstrated. On physical examination, the GT had lower lameness scores than the GC. The difference between lameness scores at T4 is probably due to the ability of the therapeutic compound (CS plus GlcN) to reduce the concentration of pro-inflammatory cytokines and prostaglandins, thus decreasing pain and consequently improving the clinical presentation of lameness [12, 25]. This difference was not more evident in the statistical analysis probably because the lameness scores ranged only from 0 to 5, with very close values and milder presentations at the end of this study. As lameness scores were close to 1 after T2 for both groups, a more sensitive lameness grading score, ranging 0 to 10, for example, could result in significant differences. Despite this, there was an important disparity in movement dynamics observed during the physical examination between groups, which was confirmed by the motion sensors (Lameness Locator), as similarly reported in other studies [8, 9, 14].

Both CS and GlcN are known for their ability to affect the expression of interleukin (IL)-1, IL-6, and C-reactive protein [12, 25]. GlcN is also effective in reducing nitric oxide synthesis by inhibiting the expression of induced nitric oxide synthase and is therefore an effective chronic inflammation and pain modulator [26]. Regardless of these anti-inflammatory characteristics, there is still controversy about the ability of these products to control clinical signs of osteoarthritis with oral administration, considering dose and bioavailability after ingestion [12, 27].

Despite the clinical improvement in GT, there was no increase in the synovial glycosaminoglycans (CS and hyaluronic acid), with no difference between groups. The possible explanations are the partial electrophoresis analysis, since the mass assessment of hyaluronic acid was not performed, and hyaluronic acid has different characteristics depending on its molecular mass [9, 10], and differences in this regard could have been observed. The complete electrophoresis analysis was not performed due to insufficient synovial fluid samples. The other explanation consists of the lack of a treated group without induced osteoarthritis. Since this treatment increases the production of chondral extracellular matrix and support the normalization of joint homeostasis [8], and observing the improvement in GT, it can be suggested that the surgically induced osteoarthritis may have influenced the measurement of synovial glycosaminoglycans. Previous studies [9, 10] reported a systemic distribution of CS and GlcN after oral administration in doses even lower than the one used in this experimentation, through urinary excretion and measurement of glycosaminoglycans in the synovial fluid, indicating that, although not verified in this study, the systemic distribution probably also occurred. Likewise, their anti-inflammatory potential has also been reported [16].

The improvement shown on physical examination was reinforced by the lower values for maximum metacarpophalangeal joint angulation in the GT. Although these values were not statistically significant, in clinical practice, even minor angle variations of 2° to 3° degrees can demonstrate joint injury or flexion restriction. Therefore, this variation demonstrates a better condition of the joint in the GT [6, 18]. GlcN and CS have been widely used for the treatment of rheumatoid arthritis in humans with favorable results [11, 26]. GlcN acts on chondrocytes and synoviocytes, inhibiting the production of molecules, such as PGE2, IL-1, and metalloproteinases, consequently modulating the inflammatory process [16, 26]. This treatment was also reported to decrease collagen degradation, thus decreasing pain, and increasing joint mobility [11, 15], which corroborates the results described here.

Biomarkers are reliable to determine characteristics that are objectively measured and/or evaluated, indicating biological or pathogenic processes or responses to a therapeutic intervention [4]. CTX II has been used as a biomarker of joint degradation and can be correlated with radiological degrees and clinical scores, being effective in monitoring disease progression and turnover of type II collagen [15, 28]. A study [15] first published the use of CTX II to monitor experimental osteoarthritis and GlcN for its treatment, reporting increased CTX II after osteoarthritis induction in rats through an anterior cruciate ligament transection, as observed in the GT and GC during the 4 time-points after arthroscopy. However, unlike this previous study [15], there was no difference between groups after treatment.

Other study [16] also reported a decreased concentration of a biomarker of collagen type II degradation (C2C) after treatment with GlcN in horses challenged with intra-articular lipopolysaccharide, differing from the results of the present experimentation, in which the performed chondral defect according to the technique described for chondral groove [21], may have had a stronger impact on chondral integrity and joint homeostasis. Both previous studies [15, 16] used techniques in which the chondral structure was not directly damaged. In the present study, the chondral defect was created with surgical instruments and the oral treatment at the dose used may not have been effective to decrease or inhibit extracellular matrix degradation with the type of induction used. However, as preventive and support treatment this compound is may be important, considering the other beneficial effects demonstrated from T0 to T4 in the GT and also reported in the literature [8–10, 12, 25].

Regarding the intra-articular inflammatory process, PGE2 presented a clear concentration peak between T1 and T2 in the GC, and PGE2 remained more constant in the GT, which was demonstrated by a lower mean concentration than that in the GC. This PGE2 biomarker result was probably due to the ability of CS and GlcN to decrease the concentration of pro-inflammatory cytokines and prostaglandins [11, 16], partially suppressing the inflammatory response in the GT, mainly in the period immediately after chondral injury.

Similarly, GT ultrasonographic examinations showed less frequent findings and scores consistent with synovitis and vascularization, in addition to a lower osteochondral score, which means a lower degree of inflammation and greater preservation of the joint related to lower PGE2 values in this group. These findings can be justified because CS and GlcN have a chondroprotective effect and reduce the catabolic processes of articular cartilage, consequently reducing the progression and clinical signs of osteoarthritis [10, 12]. The radiographic examination results can be explained by the lower sensitivity of this diagnostic modality, with significant changes only among the time-points of the study. Therefore, the analysis and scoring methodology used may not have been sensitive enough to detect minor differences between groups [6].

## Conclusion

The parameters that demonstrated the beneficial effect of using the product were the visual analysis of lameness according to the AAEP scores, determination of synovial concentrations of the biomarker PGE2, and ultrasonographic examination. Furthermore, although there was no statistically significant difference, the GT presented the best results with respect to joint flexion angle, lameness analysis through motion sensors, and histopathological analysis of the chondral repair tissue. The treatment was probably effective in modulating experimental model of osteoarthritis, with significant improvement of some parameters in the GT. However, this study demonstrated in the GT no more than a clinical mitigation of joint outcomes after induction of osteoarthritis, mainly at the end of the experiment. Thus, at the dosage administered, the oral compound was not effective to reduce the concentration of a type II collagen degradation biomarker in the synovial fluid and, therefore, did not significantly improve the tissue condition at the end of the experiment. This type of therapeutic intervention is beneficial to modulate or prevent the disease outcomes, modify the inflammatory process, and reduce the onset of clinical signs, but not effective enough to reduce the catabolic process or induce joint cartilage repair in situations in which chondral damage is significant, as in this experimental model.

## Methods

This study was approved by the Ethics Committee on Animal Use of the School of Veterinary Medicine and Animal Science at the University of São Paulo under protocol number 4119210917.

### Experimental animals

This study included 16 clinically healthy horses, without lameness and with both metacarpophalangeal joints free of abnormalities, as verified by clinical examination with lameness and flexion tests, and previous ultrasonographic and radiographic joint examinations. All included animals were adult Pure Blood Lusitano mares aged between 3 and 6 years and weighing between 450 and 530 kg. Laboratorial evaluations were also performed to confirm the participants health. The mares were randomized with criteria based on body weight and age, divided into two homogeneous groups of eight animals each, one treated and the other untreated.

The mares were fed coast-cross grass hay and commercial horse feed at a proportion of 1% of body weight and remained in stalls throughout the experiment. After a minimum adaptation period of 15 days, the metacarpophalangeal joint of the left thoracic limb in each of the 16 mares was approached dorsally by arthroscopy to create total chondral defects and induce osteoarthritis.

### Osteoarthritis induction by arthroscopy and macroscopic joint examination

The first arthroscopic procedure was defined as the time zero (T0) of the experiment, and just the left metacarpophalangeal joints of the 16 experimental animals were approached. Chondral defects or grooves, adapted from previous study [21], were created to induce osteoarthritis, with the animals in the supine position and under general inhalation anesthesia. The mares’ feeding was suspended for 12 hours before general anesthesia and they were deprived of water for 2 h. Anesthetic protocols were standardized. All mares received a pre-anesthetic treatment (10% xylazine 1.0 mg/kg, IV - Xilazin – Syntec, Brazil), 15 minutes before anesthetic induction. Diazepam (0.05 mg/Kg, IV - Compaz, Cristália, Brazil), Guaiacol Glyceryl Ether 10% (100 mg/Kg, IV; GGE - JA Saúde Animal, Brazil) and ketamine 10% (2.0 mg/Kg, IV, Cetamin-Syntec, Brazil) were used to induction, thus enabling orotracheal intubation. Anesthesia was maintained with isoflurane vaporized in 100% oxygen.

**Fig. 5:**
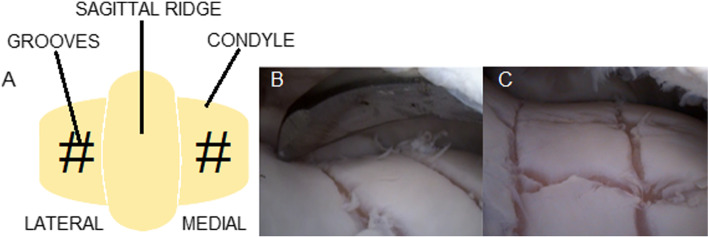
Osteoarthritis induction by arthroscopy and macroscopic joint examination. **A** Schematic demonstration of the location of the grooves (#) made in the cartilage tissue of the third metacarpal condyles to induce osteoarthritis at T0 [21]. **B** Arthroscopic image showing the position of the arthroscopic drill to make the grooves. **C** Arthroscopic image showing the final aspect of the lesions in the medial condyle at T0

An angled drill assisted by arthroscopy was used to create four lines of grooves in perpendicular directions (lateral-medial and distal-proximal), with an approximate depth of 3 mm, until reaching and exposing the subchondral bone on the medial and lateral dorsal surface of both condyles of the third metacarpal bone (Fig. 5). A macroscopic evaluation at T0 was performed immediately after the arthroscopic approach of the joint before the grooves were created. The joint capsule was closed with Caprofyl 0, and the skin closure was performed with Nylon 0 and Sultan pattern. The entire procedure was recorded. At day 120, arthroscopy was performed again for macroscopic evaluation of the joint and collection of material from synovial membrane and cartilage for microscopic analysis. The mares were checked daily to maintain their welfare, monitoring physical examination variations, and examining the surgery site. All mares received a dose of 22 mg / kg (TID, for 3 days) of sodium dipyrone in the postoperative period to prevent pain and discomfort. Antibiotic therapy with gentamicin at a dose of 6.6 mg / kg once daily was maintained until the 5th postoperative day.

### Treatment

The eight animals in the treated group (GT) were administered 10 g (1.9 g of CS and 4 g of GlcN) of the combined CS and GlcN commercial compound orally on the same day of the arthroscopy, at T0, every 12 hours for 90 days. After this period, the GT animals were administered a maintenance dose of 10 g every 24 hours for another 30 days. This is a commercial product formulation, and the dose was administered according to the manufacturer’s recommendation. The commercial product was checked, weighed on a precision scale, and mixed with pelleted feed. Both groups received commercial feed twice a day; however, only the GT received the compound. The powder formulation of the commercial oral compound consisted of C4S A (19.0 g), GlcN (40.0 g), methylsulfonylmethane (20.5 g), and excipient (100.0 g). GC group was not treated, receiving only pelleted feed.

### Physical examination

The animals in both groups, GT and GC, were clinically evaluated at T0, T1, T2, T3, and T4 (0, 30, 60, 90, and 120 days, respectively) for left forelimb lameness graded from 0 to 5 according to the American Association of Equine Practitioners (AAEP) Lameness Scale. The Lameness Locator® (Equinosis, USA) motion sensor and software were used during these evaluations for greater examination accuracy and to confirm the degree of lameness, through the Q-score result. In addition, the joint angle was measured in degrees, according to the method by Silva et al. [6], using a goniometer with the joint flexed to the physical limit or until observation of painful sensitivity.

### Synovial fluid collection and evaluation

Synovial fluid was collected from T0 to T4 from the left metacarpophalangeal joints of each animal in the GT and GC. The samples were immediately centrifuged at 1500 g for 10 minutes at 4 °C and the supernatant was stored at − 80 °C for further evaluation, using commercial enzyme-linked immunosorbent assay kits, of the synovial biomarkers C-telopeptide of type II collagen (CTX II) (Horse Cross-Linked C-Terminal Telopeptides of Type II Collagen; MyBioSource - MyBioSource Inc. San Diego, USA) and prostaglandin E2 (PGE2) (Prostaglandin E2 ELISA Monoclonal kit; Cayman Chemical - Cayman Chemical Michigan, USA) and electrophoresis of glycosaminoglycans (CS and hyaluronic acid).

### Ultrasound examination

All left metacarpophalangeal joints in the GT and GC were considered in the study and evaluated by ultrasound from T0 to T4. The ultrasound evaluation considered all articular faces, with the transducer in both longitudinal and transversal positions and with the joint in support and flexion positions. All images were stored in a digital system for subsequent evaluation. The ultrasound examinations were analyzed by two evaluators who were not aware of the study groups, according to a predefined scoring chart [18] (Table 3). The final sum of the scores for each parameter was considered.

### Radiographic examination

All left metacarpophalangeal joints considered in the study (GC and GT) were radiographically evaluated from T0 to T4. This evaluation considered five projections: lateromedial, palmar dorsal, dorsolateral-palmar medial, dorsomedial-palmar lateral, and flexed lateromedial. All images were stored in a digital system for subsequent evaluation. The radiographic examinations were analyzed by two evaluators who were not aware of the study groups, according to a predefined scoring chart [6] (Table 5). The final sum of the scores for each parameter was considered.

### Macroscopic and histopathological evaluation of the joint

The joint surfaces were macroscopically evaluated by one surgeon who were not aware of the study groups, and cartilage and synovial membrane samples were collected from the GC and GT during the arthroscopy on day 120 (T4). Global macroscopic changes were evaluated according to OARSI guidelines, through predefined macroscopic scoring chart, considering the color of synovial villi, volume and number of synovial villi and articular surface aspect [6, 19].

The collected samples were preserved in formaldehyde and processed for the subsequent preparation of histological slides, which were stained with hematoxylin and eosin. The histopathological changes at T4 were evaluated by two evaluators who were not aware of the study groups and according to the scoring described in Table 6, using scores and determinations defined by the Osteoarthritis Research Society International (OARSI) [19].

### Statistical analyses

The data were descriptively analyzed through an estimate of the mean, median, standard deviation, and interquartile range of the variables in each group and at each time-point. The Shapiro-Wilk test for normal distribution was then performed, and a nonparametric approach was chosen (Shapiro-Wilk *p*-value < 0.05). The tests were considered significant when *p* < 0.05 and analyzes were performed in SPSS 21.0 [29]. The microscopy (cartilage and synovial membrane) and macroscopic assessment were evaluated just at T4, consequently, differences between groups were verified through Mann-Withney U test.

Since the purpose of this study was the evaluation of intra-group (repeated measure - time-points T0 to T4) and inter-group (considering 2 evaluation groups) differences, a nonparametric approach, analogous to the two-way analysis of variance (ANOVA) was used with “nparLD” package in R environmental [30]. Post-hoc tests with necessary adjustments and corrections between groups were performed with Mann-Withney U test, and among time-points with Nemenyi test, when two-way ANOVA was significant. The results were presented in tables and boxplot graphs. The power (1 – β) analysis of the sample size was performed in the software GPower (2007), considering these statistical treatments. The parameters used in this analysis were: minimum expected difference between groups, error (α), power (1 – β), number of groups and number of repetitions. Therefore, it was observed that the power of sample size used in this study was satisfactory for the outcomes evaluated.

## Data Availability

All data generated or analyzed during this study are included in this published article and its related files.

## Abbreviations

CS: Chondroitin sulfate
GlcN: Glucosamine
GT: Treated group
GC: Control group
T: Time
AAEP: American Association of Equine Practitioners
CTX II: C-telopeptide of type II collagen
PGE2: Prostaglandin E2
Il-1: Interleukin1
